# Association between breakfast frequency, community space environment, and psychological symptoms: a cross-sectional study of Chinese adolescents

**DOI:** 10.3389/fpsyg.2026.1871118

**Published:** 2026-08-26

**Authors:** Xiaofei Bai, Cunbi Bi, Jian Dong, Jianzhong Sun, Qin Yang, Tao Sun

**Affiliations:** 1 Department of Physical Education, Hebei Institute of Mechanical and Electrical Technology, Xingtai, China; 2 School of Design, Zhejiang Vocational Academy of Art, Hangzhou, Zhejiang, China; 3 School of Physical Education, Anqing Normal University, Anqing, China; 4 School of Physical Education, Chizhou University, Chizhou, China; 5 School of Physical Education, Anhui Normal University, Wuhu, China

**Keywords:** adolescents, breakfast frequency, community space environment, cross-sectional study, psychological symptoms

## Abstract

**Background:**

Mental disorders among adolescents are a key global public health issue. This study aims to investigate the association between breakfast frequency and the community's space environment to promote the mental health of adolescents in China.

**Methods:**

This study employed a stratified random cluster sampling method in 2025; a total of 45,404 adolescents aged 12–17 were recruited from six regions in China. The researchers assessed the participants‘ breakfast frequency, the space environment of their neighborhoods, and their psychological symptoms. Using methods such as *t*-tests, chi-square tests, binary logistic regression, and binary logistic regression with interaction terms, we examined the joint association between adolescents' breakfast frequency, the spatial environment of their communities, and psychological symptoms.

**Results:**

The prevalence rates of emotional, behavioral, and social adjustment problems among Chinese adolescents are 27.6%, 26.6%, and 17.5%. The overall prevalence of psychological symptoms was 21.2%. Those who eat breakfast <1 time/week have a 2.41-fold higher risk of psychological symptoms than those who eat breakfast ≥4 times/week. Those who are dissatisfied with their community space environment are 1.71 times more likely to be at risk than those who are satisfied. Those who eat breakfast <1 time/week and are dissatisfied with their environment have the highest risk of psychological symptoms.

**Conclusion:**

Among Chinese adolescents, the less frequently they eat breakfast and the lower their satisfaction with their community space environment, the more severe their psychological symptoms are. regular breakfast consumption and improved community's space environment may be important public health strategies associated with lower levels of psychological symptoms among adolescents.

## 1 Introduction

Adolescent mental disorders constitute a core issue in global public health. In recent years, breakfast frequency and the community space environment have garnered significant international attention as modifiable factors influencing psychological symptoms, approximately 13% of adolescents aged 10–19 worldwide experience physical and psychological symptoms to varying degrees (Xie et al., 2023; Kieling et al., 2024). Studies have shown that poor-quality living environments (Fleckney and Bentley, 2021) and irregular breakfast habits (Zahedi et al., 2022) are associated with psychological issues such as anxiety and depression. The situation in China is equally grim; the prevalence of depressive symptoms among adolescents in areas with inadequate community public facilities is approximately 29.2% higher than in areas with adequate facilities (Xiang et al., 2022). Adolescents who do not eat breakfast regularly are 1.43 times more likely to experience anxiety symptoms than those who eat breakfast regularly (Wang H. et al., 2025). This suggests that integrating the analysis of their combined association with mental health and their independent contributions can help in developing more precise and cost-effective intervention strategies.

The community space environment and dietary habits are central components of daily health practices. Research has shown that the factors influencing psychological symptoms in adolescents are multifaceted and are closely associated with social difficulties and behavioral problems, which suggests a potential association with poor neighborhood environments and irregular breakfast habits. The community space environment refers to the physical and social conditions that influence the daily lives of young people, encompassing multiple dimensions such as safety, public services, and neighborhood support (Alderton et al., 2019). Among teenagers in North America, perceptions of community safety and neighborhood social cohesion have independent protective effects against depressive symptoms. Moreover, this effect remains robust even after adjusting for household socioeconomic status (Gepty et al., 2023). The frequency of breakfast reflects eating habits and helps regulate cognitive function and emotional wellbeing. Findings based on data from Chinese adolescents indicate that healthy eating patterns are significantly associated with a lower risk of psychological symptoms (Xu et al., 2019). This indicates that the community space environment and daily routines are closely linked to the psychological symptoms in adolescents.

Focusing on a single factor, numerous studies have examined the association between breakfast frequency, a typical variable of adolescents‘ dietary behavior, and psychological symptoms. In terms of international research, based on a large-scale study in South Australia, Burnell et al. found that people who skip breakfast score lower on measures of optimism and wellbeing than those who eat breakfast regularly (Burnell et al., 2025). Pengpid et al. observed in a sample of British university students that people who regularly skip breakfast are associated with various health risks and adverse psychological outcomes (Pengpid and Peltzer, 2020). Domestic data in China also point to a similar pattern. In their longitudinal analysis of multiethnic adolescents, Xu et al. noted that consistently skipping breakfast is associated with an increased risk of depression later in life, a trend that may be further exacerbated by late-night snacking (Xu et al., 2025a). Li et al. found, based on a study of 11,887 students aged 11–19 in Ningbo, that skipping breakfast and insufficient sleep independently and in combination increase the risk of depressive symptoms; adolescents who engage in both behaviors face a risk 4.39 times higher than those who eat breakfast regularly and get enough sleep (Li et al., 2024). On this basis, after combining multiple observational studies, Zahedi et al. proposed that skipping breakfast could serve as an independent risk marker for mental sub-healthy (Zahedi et al., 2022). Based on these findings, eating breakfast regularly is a fundamental and robust daily protective factor for adolescents' mental health. Apart from the frequency of breakfast, as an external environmental factor, the impact of the community space environment on mental health is also worthy of attention.

On the other hand, the impact of the community space environment on psychological symptoms in adolescents cannot be overlooked. A pleasant community space environment, for example, well-maintained facilities, safe sidewalks, or ample green spaces, may be related to lower stress levels, better emotional regulation, and a lower the risk of developing depression and anxiety. Recent international empirical studies have corroborated this conclusion from two perspectives. In terms of the physical environment, a longitudinal study by Larsen et al. found that higher levels of neighborhood green space during childhood were associated with a decline in externalizing symptoms among adolescent boys over time, revealing the gender-specific Protective Effects of Green Space Exposure (Larsen et al., 2025); Sheng et al. used machine learning methods to demonstrate objective environmental factors such as the safety rate of community transportation facilities and the green space visibility index have an even greater impact on adolescents' physical and mental health than subjective perceptions. Furthermore, there is a non-linear threshold effect between the two. A longitudinal study by Towe-Goodman et al. of a sample from multiple U.S. states found that exposure to green spaces in residential areas was negatively associated with internalizing symptoms in early childhood, and this association varied by age. In the social environment dimension (Towe-Goodman et al., 2024), Breedvelt's et al. review systematically examines the negative association between community social cohesion and depressive symptoms in adolescents (Breedvelt et al., 2022); A systematic review by Wood, focusing on community poverty and gentrification, found that these factors have a significant lagged effect on depressive symptoms in children and adolescents, with the impact persisting into early adulthood (Wood et al., 2023); Kim further confirmed this based on a nationally representative sample in the United States, perceived community social cohesion during adolescence can significantly reduced depression, suicidal ideation, and perceived stress in early adulthood, and remains robust even after controlling for a wide range of confounding factors (Kim et al., 2024). These findings suggest that the community space environment not only influences adolescents' daily psychological state but may also affect their long-term development through cumulative effects.

Given that both breakfast frequency and community space environment are independently associated with psychological symptoms, few studies have examined the combined effects of these two factors on psychological symptoms in adolescents, and the relationship between them and psychological symptoms remains unclear. This study conducted a cross-sectional assessment of the relationship between the community space environment and breakfast frequency among 45,404 adolescents aged 12–17 in China. Aiming to explore the relationship between these two factors and psychological symptoms in order to promote adolescent mental health and provide empirical evidence and a solid foundation for the prevention of mental health issues among adolescents.

## 2 Methods

### 2.1 Research subjects and operation procedures

This study employed a stratified cluster random sampling method (Wu et al., 2018). Participants were recruited from various regions across China in 2025. The participant selection process consists of three steps: First, based on the geographical distribution of Chinese cities, six regions were selected: East China, South China, North China, Central China, Southwest China, and Northwest China. From each region, one provincial capital and one non-provincial capital were selected as sample cities, resulting in a total of 12 cities. Second, five secondary schools were selected in each sample city. Finally, students in grades 7–12 at each middle school were selected as the sampling units, and classes were randomly selected by grade level, resulting in 21 classes being randomly selected from each school.

**Figure 1:**
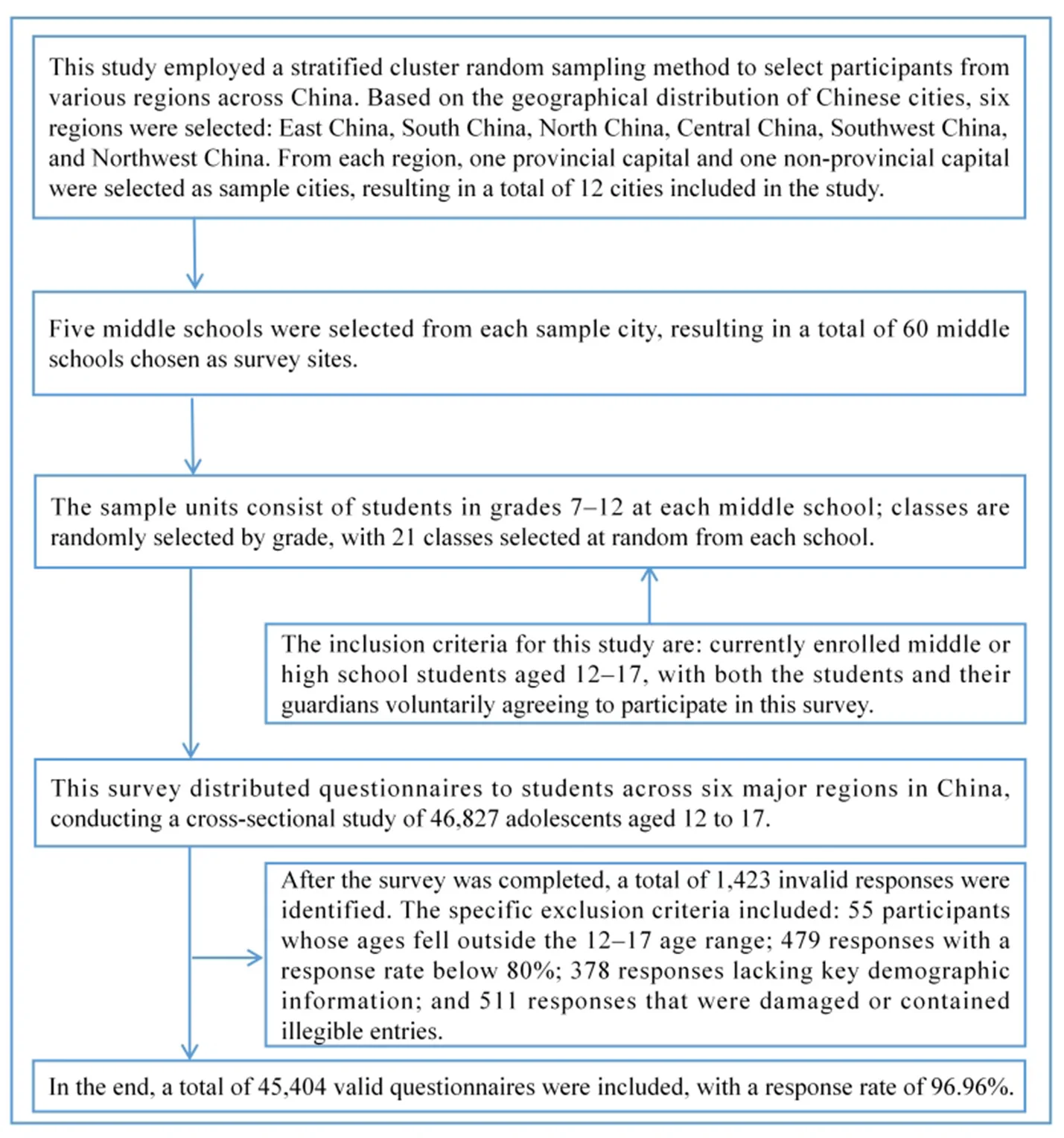
Participant extraction process for Chinese adolescents.

The inclusion criteria for this study were currently enrolled middle or high school students aged 12–17, whose participation in this survey is voluntary on the part of the student, their parents, or their legal guardians. After the investigation was completed, a total of 1,423 invalid questionnaires were identified. Specific exclusion criteria include: 55 participants were outside the 12–17 age range; 479 questionnaires had a response rate below 80%; 378 questionnaires were missing key demographic information; and 511 questionnaires were damaged or contained illegible entries. A total of 45,404 valid questionnaires were ultimately included, resulting in a response rate of 96.96%. Figure 1 illustrates the sampling process for the participants in this study.

This study was conducted in accordance with the Declaration of Helsinki. Before the evaluation, informed consent has been obtained from the parents or legal guardians. All participants voluntarily agreed to take part in this study, which was approved by the Human Ethics Committee of Anqing Normal University (AQNU2023033).

### 2.2 Breakfast frequency

This study used a single self-assessment item for evaluation, asking participants how often they eat breakfast during the week (Xu et al., 2025b). The data were collected from the question: “How often did you typically eat breakfast during the past week?” Responses are recorded using a three-point scale. These categories are: “Eat frequently” (>4 times/week), “Eat occasionally” (2–3 times/week), and “Rarely or never eat” (<1 time/week) (Wang M. E. et al., 2025). The questionnaires are distributed and collected centrally on a school-by-school basis. The survey is conducted by investigators who have undergone standardized training, and on-site instructions were provided to clarify the data entry requirements, thereby ensuring consistency in data collection standards across all schools. A single-item measure was used for breakfast frequency to minimize participant burden in this school-based survey, consistent with prior large-scale studies (Golley et al., 2017).

### 2.3 Community space environment

In this study, the community space environment is defined as the physical conditions and social characteristics in the vicinity of adolescents' daily living environments that influence their physical and mental health. The assessment is conducted using a single self-assessment item; the questionnaire asked participants: “Overall, how would you rate the quality of the space environment in the community where you currently live?” The options are categorized into three levels: “Satisfied,” “Moderate satisfaction,” and “Not satisfied.” To minimize response bias, the questionnaire instructions prompt participants to consider both physical and social factors when answering, including community health services, the extent of green space coverage, the adequacy of nighttime lighting, the safety of recreational areas, neighborly support, community safety, and the general sense of security (Pruitt et al., 2012). A single-item global measure of community space environment satisfaction was employed to facilitate efficient data collection, following the approach used in previous surveys. Although this construct is multidimensional, global satisfaction ratings have been shown to correlate strongly with multi-item environmental assessments in population-based studies (de Jong et al., 2011).

### 2.4 Psychological symptoms

This study used the Multidimensional Sub-health Questionnaire of Adolescents (MSQA) to assess the level of psychological sub-health among middle school students (Alzain et al., 2023). This tool has been used in numerous large-scale surveys in the past, and the test demonstrated good reliability and validity. The Cronbach's alpha coefficient for the full scale is 0.96, indicating a high degree of internal consistency. The scale consists of a total of 39 items, covering 18 emotional problems, 8 behavioral problems, and 13 social adjustment problems. The grading system consists of six levels, with scores ranging from 1 to 6. A score of 1 indicates that symptoms have persisted for more than 3 months, a score of 6 indicates no corresponding symptoms or a duration of less than 1 week. The lower the score, the longer the symptoms last. Drawing on the definitions used in existing research, if an individual scores 3 or less on 8 or more of the 39 items, they are deemed to have psychological symptoms. Criteria for determining mental sub-health across various dimensions: At least 3 items in the emotional problems dimension score 3 or lower, at least one item in the conduct issues dimension score 3 points or less, and at least 4 items in the social adaptation dimension score 3 or lower.

### 2.5 Covariates

The covariates included in this study were body mass index (BMI) and monthly household income. The assessment utilized the methods and tools specified in the China National Student Health Survey (CN-SSCH) (Yang et al., 2022). The participants' BMI was calculated by dividing their height by the square of their weight, specifically: BMI = Weight (kg) / Height^2^ (m^2^). The BMI index is categorized into Slimmer, Normal, Overweight, and Obese. BMI classifications for adolescents are based on the standards established by the Chinese Working Group on Obesity (WGOC): Overweight falls between the 85th and 95th percentiles, Obese above the 95th percentile (Zhang et al., 2018). The Slimmer standard is defined based on age- and sex-specific BMI cutoff values for malnutrition among Chinese adolescents (Zhang et al., 2022). Family economic level is divided into four categories: < 3000 RMB/month, 3001–5000 RMB/month, 5001–7000 RMB/month, and >7000 RMB/month (Zhou et al., 2018).

### 2.6 Statistical analysis

In this study, Continuous variables are expressed as mean ± standard deviation (M ± SD), and the results of the sub-variables are expressed as percentages (%). Chi-square tests were used to compare the prevalence of psychological symptoms among adolescents across different demographic characteristics, community space environments, and breakfast frequency. Using binary logistic regression analysis, this study evaluated the effects of the community's space environment and breakfast frequency on adolescents' psychological symptoms, as well as their emotional, behavioral, and social adjustment issues. Three models were fitted: Model 1 was unadjusted; Model 2 was adjusted for age and BMI; Model 3 was adjusted for age, BMI, and household income. During the statistical analysis phase, to further investigate the combined effect of the community space environment and breakfast frequency on psychological symptoms in adolescents, this study analyzed the data using binary logistic regression with interaction terms from the generalized linear model. The results were ultimately interpreted in terms of odds ratios (*OR*) and 95% confidence intervals (*CI*). Statistical analysis was performed using SPSS 26.0 software; the significance level is set at *P* < 0.05.

## 3 Results

This study conducted a cross-sectional survey and analysis of 45,404 Chinese adolescents aged 12–17 (22,616 males, 49.81%). The participants were 14.67 ± 1.64 years old. The study findings indicate that the overall prevalence rates of emotional problems, behavioral problems, and social adjustment problems among Chinese adolescents are 27.6%, 26.6%, and 17.5%, respectively, while the overall prevalence rate of psychological symptoms is 21.2%. With regard to gender differences, boys have higher rates of conduct issues, social adaptation difficulty, and psychological symptoms than girls, the differences were statistically significant (χ^2^ values of 58.074, 26.576, and 19.700, respectively; *P* < 0.001); However, no statistically significant gender differences were observed in emotional problems (27.8% for boys, 27.4% for girls; χ^2^ = 0.903, *P* = 0.342). Table 1 presents the basic characteristics of the Chinese adolescent participants.

**Table 1:** Basic characteristics of Chinese adolescent subjects (*N* = 45,404).

Characteristic	Boys	Girls	χ^2^/*t*-value	*p*-value	Total
	22,616	22,788			45,404
Age (M ± SD)	14.64 ± 1.62	14.70 ± 1.65	−4.232	<0.001	14.67 ± 1.64
Height (M ± SD)	169.25 ± 9.27	160.94 ± 6.47	110.798	<0.001	165.07 ± 8.42
Weight (M ± SD)	58.84 ± 13.42	51.20 ± 9.29	70.513	<0.001	55.00 ± 11.86
BMI (M ± SD)	20.40 ± 3.70	19.73 ± 3.17	20.701	<0.001	20.06 ± 3.46
Family economic level [*N* (%)]			193.84	<0.001	
<3,000 RMB/month	2,427 (10.7)	2,648 (11.6)			5,075 (11.2)
3,001–5,000 RMB/month	7,564 (33.4)	8,633 (37.9)			16,197 (35.7)
5,001–7,000 RMB/month	6,851 (30.3)	6,826 (30.0)			13,677 (30.1)
>7,000 RMB/month	5,774 (25.5)	4,681 (20.5)			10,455 (23.0)
BMI Classification [*N* (%)]			601.894	<0.001	
Slimmer	3,391 (15.0)	3,546 (15.6)			6,937 (15.3)
Normal	14,617 (64.7)	16,412 (72.1)			31,029 (68.4)
Overweight	3,188 (14.1)	2,200 (9.7)			5,388 (11.9)
Obese	1,413 (6.2)	615 (2.7)			2,028 (4.5)
Community space environment [*N* (%)]			507.625	<0.001	
Satisfied	6,619 (29.3)	8,585 (37.7)			15,204 (33.5)
Moderate satisfaction	12,066 (53.4)	11,536 (50.6)			23,602 (52.0)
Not satisfied	3,931 (17.4)	2,667 (11.7)			6,598 (14.5)
Breakfast frequency [*N* (%)]			2.942	0.23	
≥4 times/week	18,817 (83.2)	19,057 (83.6)			37,874 (83.4)
2–3 times/week	2,838 (12.5)	2,832 (12.4)			5,670 (12.5)
<1 time/week	961 (4.2)	899 (3.9)			1,860 (4.1)
Emotional problems [*N* (%)]			0.903	0.342	
No	16,321 (72.2)	16,536 (72.6)			32,857 (72.4)
Yes	6,295 (27.8)	6,252 (27.4)			12,547 (27.6)
Conduct issues [*N* (%)]			58.074	<0.001	
No	16,233 (71.8)	17,077 (74.9)			33,310 (73.4)
Yes	6,383 (28.2)	5,711 (25.1)			12,094 (26.6)
Social adaptation difficulty [*N* (%)]			26.576	<0.001	
No	18,458 (81.6)	19,017 (83.5)			37,475 (82.5)
Yes	4,158 (18.4)	3,771 (16.5)			7,929 (17.5)
Psychological symptoms [*N* (%)]			19.7	<0.001	
No	17,632 (78.0)	18,154 (79.7)			35,786 (78.8)
Yes	4,984 (22.0)	4,634 (20.3)			9,618 (21.2)

Table 2 presents an analysis of the characteristics of psychological symptoms and emotional, behavioral, and social adjustment problems among Chinese adolescents. The results show that significant differences were observed in emotional problems, conduct issues, Social adaptation difficulty, and psychological symptoms across sociodemographic factors, community space environments, family economic status, and BMI (*P* < 0.01). Boys have a higher prevalence of behavioral problems, social adjustment issues, and psychological symptoms, no gender differences were observed in emotional problems (*P* = 0.342); Adolescents with a family economic level of < 3,000 RMB/month and those dissatisfied with their community space environment had the highest prevalence of psychological problems across all dimensions (26.7%), and showed a negative correlation with family economic level and satisfaction with the living environment. Of all the variables, the association between breakfast frequency and psychological symptoms was the most statistically significant (psychological symptoms χ^2^ = 444.298, *P* < 0.001). The prevalence of psychological symptoms rose sharply from 19.6% to 37.6% as breakfast frequency decreased, this suggests that eating breakfast regularly is a modifiable factor with intervention value. There is a clear dose-response relationship between BMI classification and psychological symptoms, with an increasing trend as the degree of obesity increases (Slimmer: 20.1%, Obese: 27.5%).

**Table 2:** Characterization of adolescents' psychological symptoms in China [*N* (%)].

Characteristic	Emotional problems	*χ^2^*	*p*-value	Conduct issues	*χ^2^*	*p*-value	Social adaptation difficulty	χ^2^	*p*-value	Psychological symptoms	*χ^2^*	*p*-value
Sex		0.903	0.342		58.074	<0.001		26.576	<0.001		19.7	<0.001
Boys	6,295(27.8)			6,383(28.2)			4,158(18.4)			4,984(22.0)		
Girls	6,252(27.4)			5,711(25.1)			3,771(16.5)			4,634(20.3)		
Family economic level		74.429	<0.001		63.071	<0.001		114.761	<0.001		108.879	<0.001
<3,000 RMB/month	1,657(32.7)			1,579(31.1)			1,153(22.7)			1,355(26.7)		
3,001–5,000 RMB/month	4,382(27.1)			4,294(26.5)			2,790(17.2)			3,320(20.5)		
5,001–7,000 RMB/month	3,634(26.6)			3,480(25.4)			2,219(16.2)			2,733(20.0)		
> 7,000 RMB/month	2,874(27.5)			2,741(26.2)			1,767(16.9)			2,210(21.1)		
BMI Classification		31.357	<0.001		80.314	<0.001		40.614	<0.001		70.112	<0.001
Slimmer	1,860(26.8)			1,802(26.0)			1,156(16.7)			1,396(20.1)		
Nomal	8,471(27.3)			8,045(25.9)			5,346(17.2)			6,414(20.7)		
Overweight	1,553(28.8)			1,554(28.8)			967(17.9)			1,248(23.2)		
Obese	658(32.4)			691(34.1)			456(22.5)			557(27.5)		
Community space environment		251.889	<0.001		268.491	<0.001		283.834	<0.001		262.031	<0.001
Satisfied	3,899(25.6)			3,725(24.5)			2,438(16.0)			2,935(19.3)		
Moderate satisfaction	6,297(26.7)			6,075(25.7)			3,859(16.4)			4,794(20.3)		
Not satisfied	2,351(35.6)			2,294(34.8)			1,632(24.7)			1,889(28.6)		
Breakfast frequency		346.349	<0.001		390.54	<0.001		502.188	<0.001		444.298	<0.001
≥4 times/week	9,868(26.1)			9,474(25.0)			6,035(15.9)			7,428(19.6)		
2–3 times/week	1,886(33.3)			1,822(32.1)			1,263(22.3)			1,491(26.3)		
<1 time/week	793(42.6)			798(42.9)			631(33.9)			699(37.6)		

Table 3 presents the univariate analysis of breakfast frequency, community space environment, and psychological symptoms among Chinese adolescents. The overall results indicate that breakfast frequency is significantly associated with all dimensions of psychological symptoms (*P* < 0.001). As the frequency of breakfast decreases, the prevalence of emotional problems, behavioral issues, social adjustment difficulties, and psychological symptoms is on the rise. Among the general population, the prevalence of psychological symptoms among those who ate breakfast ≥ 4 times/week was 19.6%, the percentage of those eating 2–3 times/week rose to 26.3%, and those who ate < 1 time/week accounted for as much as 37.6%, showing a dose-response relationship. The prevalence of behavioral problems among boys in the lowest frequency group was 43.6%, the highest among all subgroups. The prevalence of psychological symptoms across all psychological dimensions was significantly higher in the group dissatisfied with their community space environment than in the satisfied group (*P* < 0.001). A gender-disaggregated analysis shows that the prevalence of psychological symptoms among female students was lowest in a supportive environment, at 18.2%. However, when moving from a generally satisfactory to an unsatisfactory environment, the increase in the prevalence of psychological symptoms among females was slightly higher than that among males, indicating that environmental satisfaction is more closely linked to psychological symptoms in females. In addition, the association between breakfast frequency and social adjustment issues was the strongest among all indicators. In the overall population, the prevalence of social adjustment problems in the < 1 time/week group was 33.9%, more than double that of the ≥4 times/week group.

**Table 3:** Univariate analysis of breakfast frequency, community space environment, and psychological symptoms among Chinese adolescents.

Variable	*N*	Emotional problems			Conduct issues			Social adaptation difficulty			Psychological symptoms		
		*N*(%)	*χ^2^*	*p*-value	*N*(%)	*χ^2^*	*p*-value	*N*(%)	*χ^2^*	*p*-value	*N*(%)	*χ^2^*	*p*-value
Boys													
Breakfast frequency			175.185	< 0.001		176.633	< 0.001		279.952	< 0.001		222.349	< 0.001
≥4 times/week	18,817	4,947(26.3)			5,010(26.6)			3,157(16.8)			3,856(20.5)		
2–3 times/week	2,838	929(32.7)			954(33.6)			651(22.9)			752(26.5)		
< 1 time/week	961	419(43.6)			419(43.6)			350(36.4)			376(39.1)		
Community space environment			122.736	< 0.001		109.181	< 0.001		126.755	< 0.001		114.451	< 0.001
Satisfied	6,619	1,752(26.5)			1,755(26.5)			1,142(17.3)			1,371(20.7)		
Moderate satisfaction	12,066	3,166(26.2)			3,251(26.9)			2,045(16.9)			2,494(20.7)		
Not satisfied	3,931	1,377(35.0)			1,377(35.0)			971(24.7)			1,119(28.5)		
Girls													
Breakfast frequency			173.01	< 0.001		214.197	< 0.001		222.246	< 0.001		222.72	< 0.001
≥4 times/week	19,057	4,921(25.8)			4,464(23.4)			2,878(15.1)			3,572(18.7)		
2-3 times/week	2,832	957(33.8)			868(30.6)			612(21.6)			739(26.1)		
< 1 time/week	899	374(41.6)			379(42.2)			281(31.3)			323(35.9)		
Community space environment			136.466	< 0.001		145.912	< 0.001		149.779	< 0.001		144.844	< 0.001
Satisfied	8,585	2,147(25.0)			1,970(22.9)			1,296(15.1)			1,564(18.2)		
Moderate satisfaction	11,536	3,131(27.1)			2,824(24.5)			1,814(15.7)			2,300(19.9)		
Not satisfied	2,667	974(36.5)			917(34.4)			661(24.8)			770(28.9)		
Total													
Breakfast frequency			346.349	< 0.001		390.54	< 0.001		502.188	< 0.001		444.298	< 0.001
≥4 times/week	37,874	9,868(26.1)			9,474(25.0)			6,035(15.9)			7,428(19.6)		
2–3 times/week	5,670	1,886(33.3)			1,822(32.1)			1,263(22.3)			1,491(26.3)		
< 1 time/week	1,860	793(42.6)			798(42.9)			631(33.9)			699(37.6)		
Community space environment			251.889	< 0.001		269.498	< 0.001		283.834	< 0.001		262.031	< 0.001
Satisfied	15,204	3,899(25.6)			3,725(24.5)			2,438(16.0)			2,935(19.3)		
Moderate satisfaction	23,602	6,297(26.7)			6,075(25.7)			3,859(16.4)			4,794(20.3)		
Not satisfied	6,598	2,351(35.6)			2,294(34.8)			1,632(24.7)			1,889(28.6)		

Table 4 presents the results of a binary logistic regression analysis examining the effects of breakfast frequency and the community space environment on psychological symptoms among Chinese adolescents. Overall, using a breakfast frequency of ≥4 times/week as the reference, the group breakfast frequency 2–3 times/week showed a significantly increased risk of psychological symptoms (*OR* = 1.45, *95% CI*: 1.36–1.55), the risk was also significantly higher in the < 1 time/week group (*OR* = 2.41, *95% CI*: 2.19–2.66). In the community space environment, the risk of psychological symptoms was slightly elevated in the moderately satisfied group (*OR* = 1.07, *95% CI*: 1.02–1.13), and the risk was significantly higher in the dissatisfied group (*OR* = 1.71, *95% CI*: 1.60–1.83). A gender-stratified analysis showed that the risk increase associated with environmental dissatisfaction was slightly higher for female students than for male students (*OR* for females = 1.86, *OR* for males = 1.56). The incidence was significantly higher in the group that ate breakfast < 1 time/week among both boys and girls (*OR* for boys = 2.43, *OR* for girls = 2.38).

**Table 4:** A binary logistic regression for breakfast frequency, community space environment, and psychological symptoms among Chinese adolescents.

Sex/variable	Group	Model 1		Model 2		Model 3	
		*OR (95% CI)*	*p*-value	*OR (95% CI)*	*p*-value	*OR (95% CI)*	*p*-value
Boys							
Breakfast frequency	≥4 times/week	1.00	–	1.00	–	1.00	–
	2–3 times/week	1.40 (1.28–1.53)	< 0.001	1.41 (1.28–1.54)	< 0.001	1.39 (1.27–1.53)	< 0.001
	< 1 time/week	2.49 (2.18–2.85)	< 0.001	2.47 (2.16–2.82)	< 0.001	2.43 (2.12–2.78)	< 0.001
Community space environment	Satisfied	1.00		1.00		1.00	
	Moderate satisfaction	1.00 (0.93–1.07)	0.944	1.00 (0.93–1.07)	0.932	1.00 (0.93–1.08)	0.918
	Not satisfied	1.52 (1.39–1.67)	< 0.001	1.52 (1.39–1.67)	< 0.001	1.56 (1.42–1.71)	< 0.001
Girls							
Breakfast frequency	≥4 times/week	1.00	–	1.00	–	1.00	–
	2–3 times/week	1.53 (1.40–1.68)	< 0.001	1.53 (1.39–1.67)	< 0.001	1.51 (1.38–1.66)	< 0.001
	< 1 time/week	2.43 (2.11–2.80)	< 0.001	2.41 (2.09–2.78)	< 0.001	2.38 (2.07–2.75)	< 0.001
Community space environment	Satisfied	1.00		1.00		1.00	
	Moderate satisfaction	1.12 (1.04–1.20)	0.002	1.12 (1.04–1.20)	0.002	1.12 (1.05–1.21)	0.002
	Not satisfied	1.82 (1.65–2.01)	< 0.001	1.84 (1.66–2.03)	< 0.001	1.86 (1.68–2.06)	< 0.001
Total							
Breakfast frequency	≥4 times/week	1.00	–	1.00	–	1.00	–
	2–3 times/week	1.46 (1.37–1.56)	< 0.001	1.47 (1.37–1.56)	< 0.001	1.45 (1.36–1.55)	< 0.001
	< 1 time/week	2.47 (2.24–2.72)	< 0.001	2.44 (2.22–2.69)	< 0.001	2.41 (2.19–2.66)	< 0.001
Community space environment	Satisfied	1.00	–	1.00	–	1.00	–
	Moderate satisfaction	1.07 (1.01–1.12)	0.015	1.07 (1.01–1.12)	0.016	1.07 (1.02–1.13)	0.01
	Not satisfied	1.68 (1.57–1.79)	< 0.001	1.68 (1.57–1.79)	< 0.001	1.71 (1.60–1.83)	< 0.001

Model 1: unadjusted; Model 2: adjusted for age and BMI; Model 3: adjusted for age, BMI, and household income.

**Figure 2:**
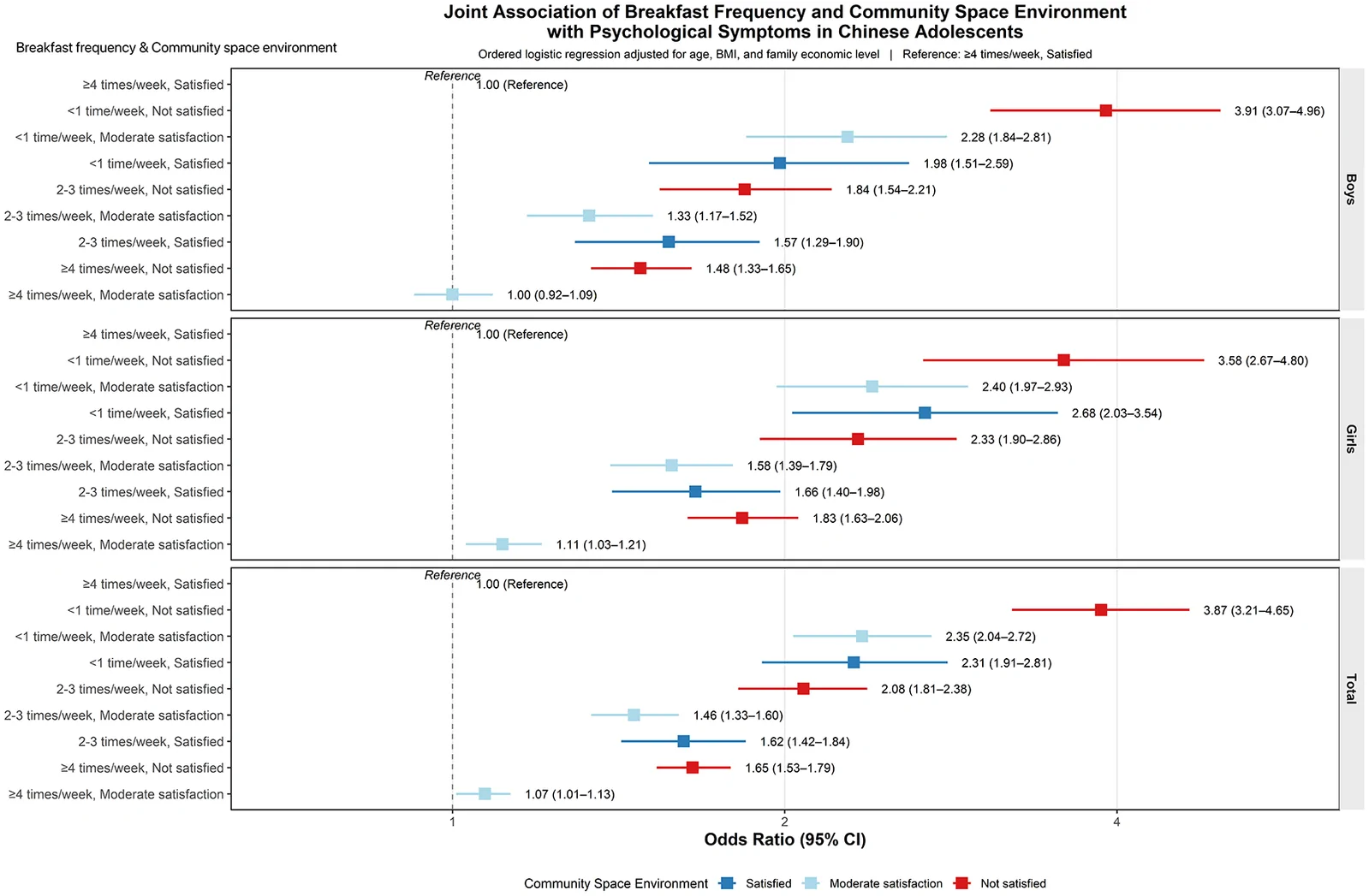
Trends in ORs of binary logistic regression with interaction terms analysis of breakfast frequency, community space environment, and psychological symptoms in Chinese adolescents.

Table 5 presents the binary logistic regression with interaction terms analysis of breakfast frequency, community space environment, and psychological symptoms among Chinese adolescents. The model accounted for variables such as age, body mass index (BMI), and family economic level. Overall, using adolescents who ate breakfast ≥4 times/week and are satisfied with their community space environment as the reference group, as the breakfast frequency decreases and satisfaction with the community space environment declines, the risk of psychological symptoms among adolescents gradually increases. The group that ate breakfast at least four times a week and reported satisfaction with their community space environment had the lowest risk of psychological symptoms, compared with the reference group; the risk of having breakfast < 1 time/week and being dissatisfied with the community's space environment was 3.87 times higher in this group (*95% CI*: 3.24–4.69, *P* < 0.001). However, in the analysis stratified by gender, among female students, the risk of psychological symptoms was significantly higher when breakfast frequency was < 1 time/week and satisfaction with the community space environment was high (*OR* = 2.68, *95% CI*: 2.03–3.54, *P* < 0.001). Figure 2 shows the trend in the OR value.

**Table 5:** Binary logistic regression with interaction terms analysis of breakfast frequency, community space environment, and psychological symptoms among Chinese adolescents.

Sex	Breakfast frequency	Community space environment	*OR (95% CI)*	*p*-value
Boys	≥4 times/week	Satisfied	1.00	–
		Moderate satisfaction	1.00 (0.923–1.088)	0.956
		Not satisfied	1.48 (1.335–1.647)	< 0.001
	2–3 times/week	Satisfied	1.57 (1.291–1.898)	< 0.001
		Moderate satisfaction	1.33 (1.168–1.519)	< 0.001
		Not satisfied	1.84 (1.541–2.206)	< 0.001
	< 1 time/week	Satisfied	1.98 (1.507–2.593)	< 0.001
		Moderate satisfaction	2.28 (1.845–2.806)	< 0.001
		Not satisfied	3.91 (3.071–4.965)	< 0.001
Girls	≥4 times/week	Satisfied	1.00	–
		Moderate satisfaction	1.11 (1.028–1.205)	0.008
		Not satisfied	1.83 (1.633–2.058)	< 0.001
	2–3 times/week	Satisfied	1.66 (1.395–1.982)	< 0.001
		Moderate satisfaction	1.58 (1.390–1.795)	< 0.001
		Not satisfied	2.33 (1.899–2.863)	< 0.001
	< 1 time/week	Satisfied	2.68 (2.031–3.536)	< 0.001
		Moderate satisfaction	2.40 (1.966–2.932)	< 0.001
		Not satisfied	3.58 (2.670–4.797)	< 0.001
Total	≥4 times/week	Satisfied	1.00	–
		Moderate satisfaction	1.07 (1.008–1.129)	0.026
		Not satisfied	1.65 (1.531–1.787)	< 0.001
	2–3 times/week	Satisfied	1.62 (1.422–1.844)	< 0.001
		Moderate satisfaction	1.46 (1.335–1.604)	< 0.001
		Not satisfied	2.08 (1.815–2.376)	< 0.001
	< 1 time/week	Satisfied	2.31 (1.907–2.809)	< 0.001
		Moderate satisfaction	2.35 (2.036–2.718)	< 0.001
		Not satisfied	3.87 (3.214–4.653)	< 0.001

Binary logistic regression with interaction terms model adjusted for age, BMI, and family economic level.

Model 1: unadjusted; Model 2: adjusted for age and BMI; Model 3: adjusted for age, BMI, and household income.

## 4 Discussion

This study is the first to examine a large, nationwide sample of adolescents to analyze the association between breakfast frequency, community space environment, and psychological symptoms. We found that the frequency of breakfast consumption is negatively correlated with the prevalence of psychological symptoms among adolescents. This finding is consistent with the conclusions of several previous studies (López-Gil et al., 2024; Naumoska et al., 2025). At the same time, we also observed that adolescents who were more satisfied with their community space environment had a lower prevalence of psychological symptoms. Of particular concern is that, after analyzing the data by gender, among female students, when breakfast frequency was < 1 time/week but satisfaction with the community space environment was high, the risk of psychological symptoms remained significantly elevated (*OR* = 2.68, *95% CI*: 2.03–3.54, *P* < 0.01), a similar pattern was observed among male students (*OR* = 1.98, *P* < 0.001). This indicates that under favorable environmental conditions, the adverse association between irregular breakfast habits and psychological symptoms remains independent, and a good community space environment cannot fully compensate for the risks associated with infrequent breakfasts. This combined association may operate through biological and psychosocial pathways. Irregular breakfast habits could be related to stress regulation, while poor community environments may be associated with reduced access to social and physical resources that help buffer stress. These pathways might also be linked to each other. From a public health standpoint, this implies that interventions addressing both diet and environment together may be more effective than targeting either one separately.

This study found that eating breakfast regularly is independently associated with lower odds of psychological symptoms in adolescents. After adjusting for relevant covariates, adolescents who eat breakfast less than once a week face a risk 2.41 times higher than those who eat breakfast regularly, demonstrating a clear dose-response relationship. This trend is highly consistent with the findings of previous international studies. Breakfast is an essential component of the daily diet; skipping it can disrupt energy supply and cause neuroendocrine imbalances, which may gradually lead to more persistent psychological distress (Minari and Pisani, 2025; Micha et al., 2011), ultimately affecting the central nervous system's ability to regulate emotions (Tucker et al., 2025). The nutrients provided by breakfast are difficult to fully replace through lunch and dinner alone; a prolonged deficiency can make adolescents more prone to symptoms such as anxiety and irritability when facing academic pressure and interpersonal conflicts (Sălcudean et al., 2025). In the long run, People who consistently skip breakfast are prone to developing the habit of snacking late at night, disrupting circadian rhythms and the balance of the gut microbiota, and the cumulative effect of worsening depressive symptoms (Codoñer-Franch et al., 2023). This eating pattern warrants particular attention among adolescents, as puberty itself tends to be associated with a delay in the sleep-wake cycle; when combined with the circadian disruption linked to irregular breakfast habits, this may relate to a vicious cycle of unhealthy lifestyle habits and psychological symptoms. The synergistic effects of these multifaceted factors are collectively associated with a lower likelihood of psychological symptoms in adolescents.

This study also found a significant association between satisfaction with the community's space environment and psychological symptoms among adolescents. Adolescents who are dissatisfied with their community space environment are 1.71 times more likely to experience psychological symptoms than those who are satisfied. Compared to objective environmental measurement indicators, subjective satisfaction provides a more comprehensive reflection of the combined effects of the physical and social environments on adolescents' psychological symptoms (Lindsley et al., 2026). The association of the community space environment with psychological symptoms is multifaceted. Research has shown that the quality of the physical environment and social networks within a community influence adolescents' emotional resilience in various ways (Shaughnessy et al., 2025). Conversely, adolescents who are dissatisfied with their community's space environment may find their outdoor activities and social interactions restricted, which may be related to increased sedentary behavior and may in turn be associated with psychological issues. The community space environment plays a crucial role in shaping adolescents' social and emotional skills. Research has shown that adolescents' perceptions of community safety and neighborhood cohesion provide an independent protective effect against depressive symptoms, and that this protective effect can extend into early adulthood (Berg, 2025; Brieant and Burt, 2025). For adolescents in a critical phase of social development, a negative community space environment can exacerbate their sense of social isolation and undermine their self-esteem, thereby triggering a range of psychological symptoms (Wade et al., 2025). This suggests that the association of the community space environment with adolescents' psychological symptoms may be related to the combined effects of behavioral constraints and a lack of social cohesion.

This study has certain strengths and limitations. As for the advantages, this study is the first to use a nationwide sample in China to analyze the association between adolescents' breakfast frequency, the space environment of their communities, and psychological symptoms, thereby providing practical guidance for the development and intervention of adolescent psychological symptoms. Second, this study has a large sample size (*N* = 45,404), covering multiple regions across China, and it has strong national representativeness. At the same time, this study also has its limitations. First, cross-sectional studies cannot establish causal relationships; future validation will require prospective cohort studies. Second, the frequency of breakfast and satisfaction with community spaces rely on self-reports, which may be subject to recall bias. The use of single-item measures may not fully capture the multidimensional nature of the community space environment. In the future, such assessments could be conducted using more objective measurement tools.

## 5 Conclusion

There is an association between breakfast frequency and the community space environment and psychological symptoms among Chinese adolescents. Regular breakfast consumption and improved community space environments may represent potential targets for future research and mental health promotion strategies.

## Data Availability

The raw data supporting the conclusions of this article will be made available by the authors, without undue reservation.
